# Behavioural Features of Cerebral Visual Impairment Are Common in Children With Down Syndrome

**DOI:** 10.3389/fnhum.2021.673342

**Published:** 2021-06-14

**Authors:** Gemma J. Wilton, Rhodri Woodhouse, Valldeflors Vinuela-Navarro, Rachel England, J. Margaret Woodhouse

**Affiliations:** ^1^School of Optometry and Vision Sciences, Cardiff University, Cardiff, United Kingdom; ^2^Optometry and Vision Science Research Group, Optometry School, Aston University, Birmingham, United Kingdom

**Keywords:** Down syndrome, cerebral visual impairment, CVI, visual perception, refractive error, dorsal stream, ventral stream

## Abstract

It is widely recognised that children with Down syndrome have a broad range and a high prevalence of visual deficits and it has been suggested that those with Down syndrome are more likely to exhibit visual perception deficits indicative of cerebral visual impairment. This exploratory study aims to determine the prevalence of behavioural features suggestive of cerebral visual impairment (CVI) occurring with Down syndrome and whether the visual problems can be ascribed to optometric factors. A cohort of 226 families of children with Down syndrome (trisomy 21), aged 4–17, were invited to participate in a validated question inventory, to recognise visual perception issues. The clinical records of the participants were then reviewed retrospectively. A five-question screening instrument was used to indicate suspected CVI. The majority of the 81 families who responded to the questionnaire reported some level of visual perceptual difficulty in their child. Among this cohort, the prevalence of suspected CVI as indicated by the screening questionnaire was 38%. Only ametropia was found to have a significant association with suspected CVI, although this increased the correct prediction of suspected CVI outcome by only a small amount. Results suggest that children with Down syndrome are more likely to experience problems consistent with cerebral visual impairment, and that these may originate from a similar brain dysfunction to that which contributes to high levels of ametropia and failure to emmetropise. It is important that behavioural features of CVI are recognised in children with Down syndrome, further investigations initiated and appropriate management applied.

## Introduction

This study set out to estimate the prevalence of behavioural features associated with cerebral visual impairment (CVI) among children with Down syndrome (DS) and to identify whether a child's reported behavioural features are related to optometric deficits. This information could help better understand a child's needs and tailor more appropriate and accessible educational strategies.

CVI is one of the most common causes of visual impairment (Kong et al., 2012; Philip and Dutton, 2014; Solebo et al., 2017), responsible for 27–48% of childhood visual impairment in developed nations (Rahi and Cable, 2003; Kong et al., 2012; Chong and Dai, 2014). Difficulties met by children with CVI vary greatly but have been shown to reduce quality of life even in less severe cases (Sakki et al., 2021). Problems arising from CVI in children without DS have been related to the widely accepted model of two visual pathway streams. Damage to the posterior parietal lobes affects dorsal stream functions such as processing the whole visual scene, visually guided movement and perception of motion. This can cause difficulty handling crowded scenes, difficulty seeing moving objects, impaired visual attention and difficulty negotiating steps and uneven flooring (Dutton and Jacobson, 2001; Dutton et al., 2004; Dutton, 2010). Damage to the temporal lobes affects ventral stream functions and results in difficulties with recognition, manifesting as difficulties recognising faces, facial expressions, objects or abstract drawings and difficulty with orientation and route finding (Dutton and Jacobson, 2001; Dutton et al., 2004; Dutton, 2010).

Common causes of CVI include hypoxia, brain injury or infection, metabolic disorders, seizure disorders and *in utero* drug exposure (Philip and Dutton, 2014; Pehere et al., 2018). For many children, visual impairment occurs as a trait of, or in conjunction with, a multitude of complex systemic diseases (Flanagan et al., 2003; Rahi and Cable, 2003; Bodeau-Livinec et al., 2007; Rahi et al., 2009). It has been suggested that there may be an association between CVI and DS (Little et al., 2007, 2009).

CVI can be difficult to diagnose as its symptoms can exist in varying combinations and severities (Dutton, 2010) and many of its characteristics can overlap with other conditions. The known association between CVI and other complex systemic conditions may also present challenges in visual examination.

Children with DS are also at risk of ocular pathology and vision problems such as reduced best corrected visual acuity, poor accuracy of accommodation, a high incidence and magnitude of refractive error with a less successful emmetropisation process, a higher incidence of strabismus, cataract, epiphora and reduced contrast sensitivity compared to typically developing children (Courage et al., 1994; Cregg et al., 2001, 2003; Stephen et al., 2007; Zahidi et al., 2018).

## Materials and Methods

A total of 221 children with DS aged 4–17 years were invited to take part; 37 when attending the School of Optometry & Vision Sciences to participate in ongoing research (for which written consent was obtained), and 184 who attended the School clinic between 1st November 2016 and 1st November 2018, by means of a letter sent to parents. Parents were invited to complete an online questionnaire, and its completion was taken as consent for the study.

### CVI Criteria

The online questionnaire was created using the 51-question inventory by Dutton (2010), that explores difficulties experienced by children in everyday tasks that are vision-dependent. The questionnaire used a scale of qualitative responses; “Never” (score (1), “Rarely” (2), “Sometimes” (3), “Often” (4), and “Always” (5). An option of “Not Applicable” was available and if selected, was removed from the analysis. A positive result (a score of 4 or 5) on three or more of questions 2, 18, 19, 24, and 27 was used as a positive screening for suspected CVI. This 5-question screening tool was determined by those difficulties commonly reported in children with CVI and rarely in those without (Dutton, 2010) and is a reliable diagnostic screening tool (Macintyre-Beon et al., 2012; Philip et al., 2016) with a good construct validity; sensitivity of 81.7% and specificity of 87.2% (Gorrie et al., 2019).

### Optometric Data

Retrospective review of participants' clinical records was conducted and eight factors which could impact on the incidence of behavioural features of CVI were identified: age, gender, corrected visual acuity (binocular LogMAR), ametropia (best vision sphere of the least ametropic or fixing eye), magnitude of astigmatism (of least astigmatic or fixing eye), strabismus (present or not), accommodation (accurate or not), nystagmus (present or not).

### Ethical Approval

This study was part of a wider longitudinal study in children with DS and had ethical approval from the National Institute for Social Care and Health Research Ethics Service (08/MRE09/46, amendment 5, 7th July 2016).

## Results

### Population Characteristics

A response rate of 36.7% was achieved; 81 responders. The gender and age of the respondents were compared to those of all invited participants. Gender (55.6% female) and age (4.4–17 years, mean 9.9 years) did not differ significantly from the invited population (χ^2^ = 0.86, *p* > 0.05 and t = −1.53, *p* > 0.05, respectively).

Participants had a mean binocular visual acuity of 0.29 LogMAR (standard deviation, SD = 0.19), recorded using a variety of tests, based on the child's age and abilities: Cardiff Acuity Test, Crowded or Uncrowded LogMAR Kay Picture Test, Crowded or Uncrowded LogMAR Kay Acuity Test and the Keeler Crowded or Uncrowded LogMAR Visual Acuity Test.

Ten children (12.3%) in the study population had nystagmus and 13 children (16%) manifest strabismus (10 esotropia including 6 fully accommodative, two exotropia, and one vertical tropia).

Accommodative ability was recorded using dynamic retinoscopy; 39 children accommodated accurately, 39 had under-accommodation (all wore multifocal spectacle correction) and three children had inconclusive results.

Choice of refraction method was based on clinical need: static, Mohindra or cycloplegic retinoscopy. The best vision sphere of the least ametropic eye (fixing eye in strabismus) was recorded. There was no correlation between ametropia and age; Pearson, r = 0.04; *n* = 81; *p* > 0.05 (*p* = 0.72). The distribution of refraction is shown in Table 1.

**Table 1:** The number of responders falling into each refractive error category.

**Refraction**	**Definition**	**Number *n***	**Percentage %**
			**(total *n =* 81)**
Emmetropia	−0.75 D to +2.75 D	20	24.7
Hypermetropia only	> +2.75 D	13	16.0
Myopia only	>-0.75 D	1	1.2
Simple astigmatism	One meridian ametropic and the other meridian emmetropic	20	24.7
Hypermetropic Astigmatism	Both meridians hypermetropic	22	27.2
Myopic Astigmatism	Both meridians myopic	5	6.2

*The refraction is for the least ametropic or fixing eye*.

### CVI

Of the 81 children, 31 screened positive for suspected CVI; a prevalence of 38.3%. This report uses the term suspected CVI to mean a positive classification according to the five-question criteria.

### Total Score

**Figure 1:**
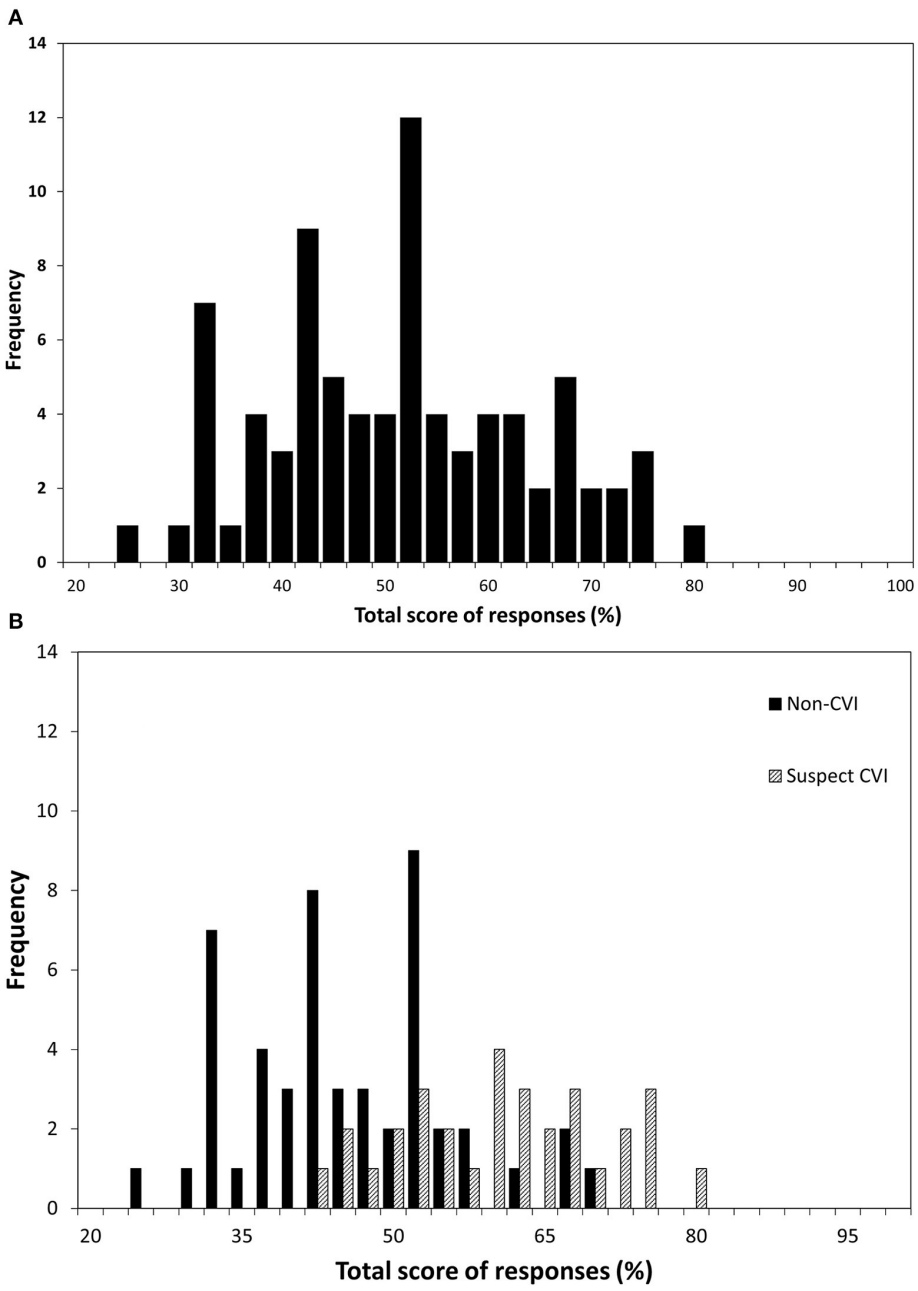
The frequency distribution of the total raw scores in 81 children. The x-axis shows total score expressed as a percentage of applicable questions for **(A)** all children and **(B)** children divided into “suspected CVI” and “non-CVI” according to whether the child screened positive on the five-question CVI screening tool.

The raw total score for each participant was attained by summing all question responses and was expressed as a percentage of the total questions applicable for that participant. Both groups were normally distributed (Figure 1). The minimum score would be 20% (every question recorded as “Never” and awarded one). No child had a minimum score. The mean total score for children with suspected CVI was 59.5% (SD = 10.5%, range = 41.2–78.8, Shapiro-Wilk *p* > 0.05) and the mean for children without suspected CVI was 44.1% (SD = 10.3%, range = 24.0–67.8, Shapiro-Wilk *p* > 0.05); the difference was significant (t = 6.286, *p* < 0.001).

### Positive Score

**Figure 2:**
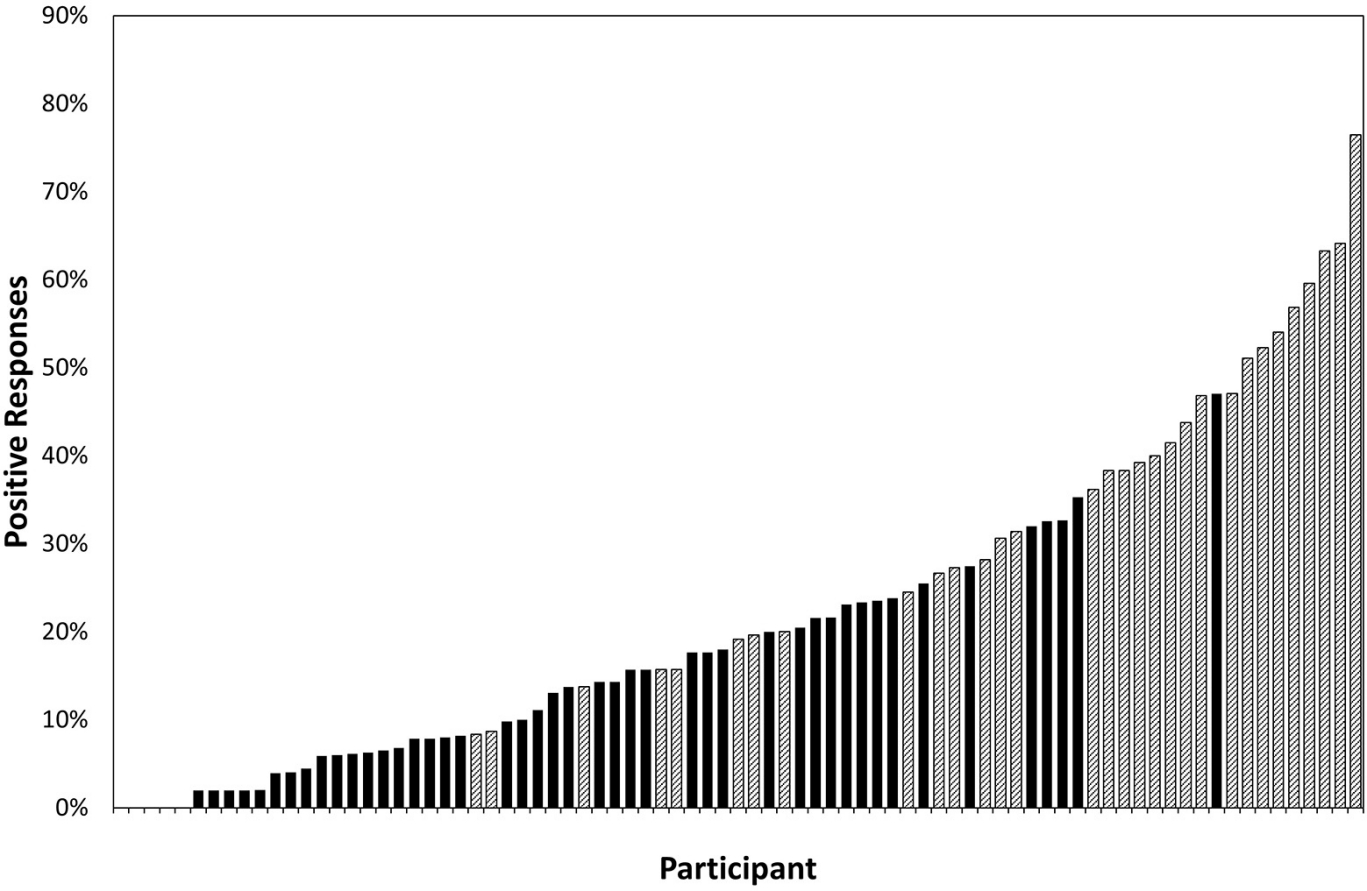
The percentage of questions (excluding those reported as “not applicable”) to which each of 81 participants responded positively (a score of four or five on the question inventory) ranked by increasing number of positive responses; crosshatching represents the participants who fitted the screening criteria for suspected CVI and solid fill represents the participants who did not.

The positive score for each participant was attained by summing the number of positive responses (Often or Always) and expressed as a percentage of the total applicable questions. Of children with suspected CVI, the mean number of positive responses was 36.7% (SD = 17.7%, range = 8.3–76.5%), compared to 13.5% (SD = 11.1%, range = 0–47.1%) among children without suspected CVI. The difference was significant (t = 7.269, *p* < 0.001). The range of positive responses is shown as a continuum in Figure 2, illustrating the large overlap between those children with and without suspected CVI.

### Individual Questions

**Figure 3:**
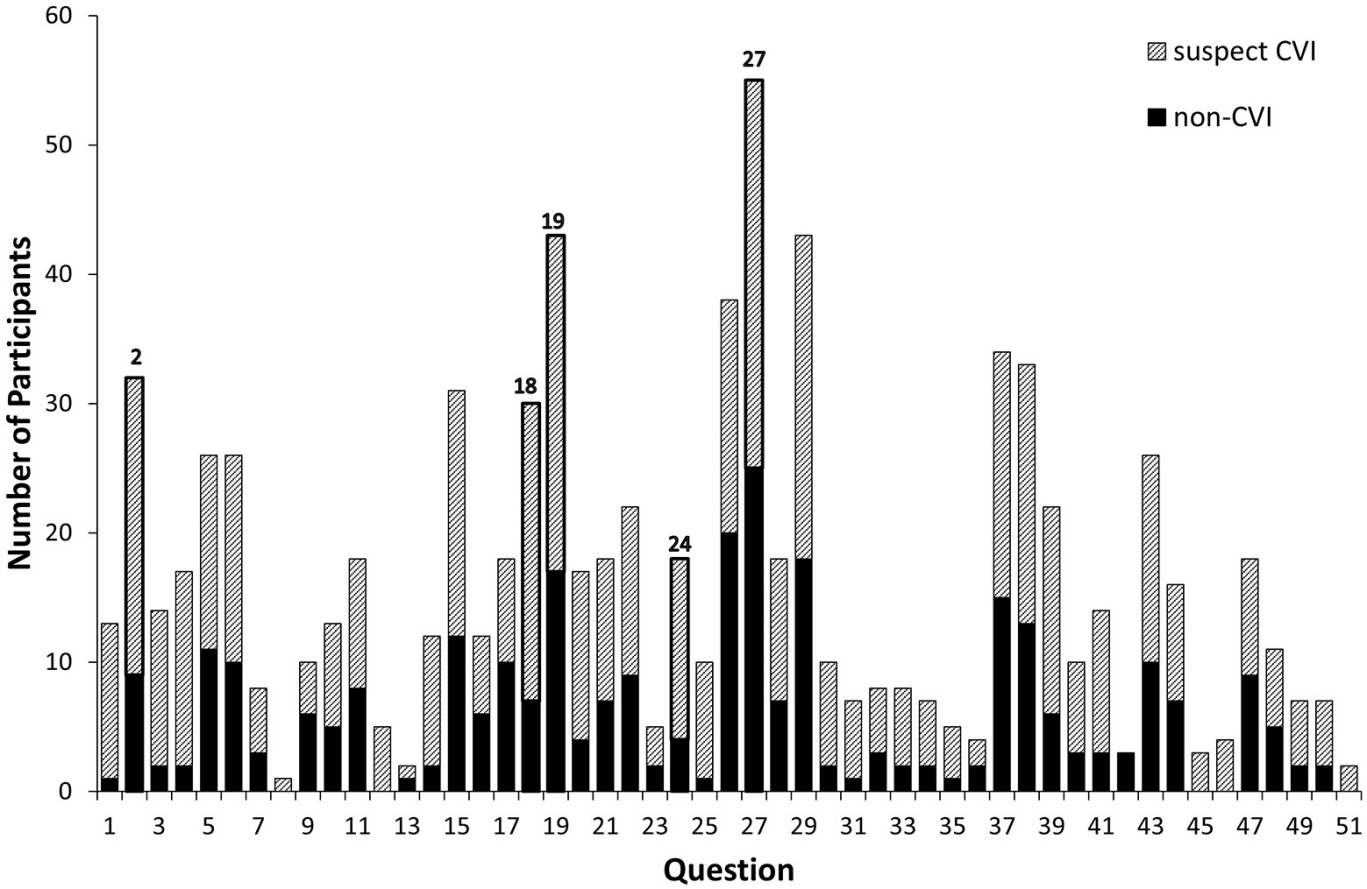
The number of participants (81 in total) who responded positively to each question; crosshatching represents the participants who fitted the screening criteria for suspected CVI and solid fill represents the participants who did not. Questions 2, 18, 19, 24, and 27 are the diagnostic questions used in the CVI screening tool and are outlined.

Figure 3 shows the number of participants whose parents responded positively to each question and therefore highlights the questions that most frequently elicited positive responses and the weighting of suspected CVI and non-suspected-CVI responses for each question.

The 10 questions most frequently eliciting positive responses (“Often” Or “Always”) were “Does your child”:

2. Have difficulty walking downstairs?

15. Have difficulty seeing things which are moving quickly, such as small animals?

18. Have difficulty catching a ball?

19. Have difficulty seeing something which is pointed out in the distance?

26. Sit closer to the television than about 30 cm?

27. Find copying words or drawings time-consuming and difficult?

29. Find uneven ground difficult to walk over?

37. Find it difficult to keep on task for more than 5 minutes?

38. After being distracted, find it difficult to get back to what he or she was doing?

43. Have difficult behaviour in a busy supermarket or shopping centre?

There were several questions that elicited positive responses equally between children who met the criteria for suspected CVI and those who did not (e.g., 47; Does your child mistakenly identify strangers as people known to them?) and some questions that were responded to positively by large numbers with and without suspected CVI (e.g., 27; Does your child find copying words or drawing time-consuming and difficult)?

There were five questions that elicited responses *only* from children with suspected CVI, although numbers were small:

8: Does your child leave food on the right or left side of their plate?

12: Does your child bump into door/frames or partly open doors?

45: Does your child have difficulty recognising close relatives in real life?

46: Does your child have difficulty recognising close relatives from photographs?

51: Does your child have difficulty recognising familiar objects, such as the family car?

### Optometric Factors and Suspected Cerebral Visual Impairment

Each factor was tested independently to determine any association with positive screening outcome for CVI. Continuous factors were tested using a Mann Whitney *U*-Test; categorical factors with a χ^2^ test. The raw data for each categorical factor were inspected for outliers. Two data points with refractive errors of −12.00 D and −7.25 D were more than 1.5 × IQR away from the mean and were removed from the analysis.

Of these eight factors, only absolute refractive error (equivalent sphere) was found to be significantly different between those with and without suspected CVI (*p* = 0.010) with two outliers removed. The higher the refractive error, the more likely was a child to screen positive for CVI. To determine whether refractive error is a suitable predictor for the incidence of suspected CVI, a univariate logistic regression was performed. The outcome of the logistic regression is given in Table 2. However, the model was only able to correctly predict suspected CVI in 62% of cases, compared to the null model's 60.8%.

**Table 2:** Outcome of logistic regression.

**Factor**	**β**	**SE β**	**Wald's** **χ**^**2**^	**df**	**p**	**Odds ratio (95% CI)**
Constant	−1.461	0.487	9.006	1	0.003	NA
Refractive error	0.340	0.137	6.104	1	0.013	1.404 (1.073–1.839)
Test			**χ**^**2**^	**df**	**p**	
Overall model			6.757	1	0.009	
Hosmer & Lemeshow (Goodness-of-fit)			8.700	7	0.275	

*All statistics were calculated using SPSS v25.0 (IBM Corp. Armonk, NY, USA). Further descriptive measures of goodness-of-fit given by Cox and Snell R^2^ = 0.082, Nagelkerke R^2^ = 0.111*.

## Discussion

In this sample of children, parents reported a high prevalence of visual perceptual problems including many that have not been previously described in Down syndrome, and that are consistent with cerebral visual impairment. A continuum of CVI-associated behavioural features was observed, in which 93.8% of children exhibited at least one CVI-associated symptom and, overall, 38.3% of children could be classified as suspected CVI, based on the five-question screening criteria. Since sampling bias is possible, the prevalence was calculated, assuming that, at one extreme, all non-responders would screen negative for CVI and, at the other extreme, all non-responders would screen positive for suspected CVI. This reveals that th prevalence of suspected CVI in a population of children with DS lies within the range of 13.7–77.9%.

Due to differing definitions and diagnostic criteria, a prevalence of CVI in a general population of children is unknown but a recent cross-sectional study using the same five screening questions suggested that at least 3.4% of mainstream primary school children (age 5–11 years old) exhibited at least one symptom of CVI (Williams et al., 2021). Another review, using a different question inventory, also showed a continuum, and visual perceptual difficulties in up to 3.9% of typical 13–14 year olds (Williams et al., 2011). The current study therefore indicates a higher risk of CVI-related difficulties among children with DS. Positive responses were given to 45 out of 51 questions, suggesting a wide range of symptoms.

The continuum of responses demonstrated in Figure 2 shows that there is no clear cut-off between children who can be deemed to have suspected CVI and those who cannot. The total score of all children with DS is normally distributed, as opposed to the skewed distribution among typical children (Williams et al., 2011) suggesting that the majority of children with DS exhibit some level of visual perceptual dysfunction.

The full 51 question inventory has been shown to elicit some positive responses from children with autism spectrum disorder (ASD) (Dutton, 2010), but the five question diagnostic criteria have been specifically chosen to represent difficulties associated with dorsal stream dysfunction and have no overlap with the Social Communication Questionnaire, designed to recognise ASD (Gorrie et al., 2019).

Over 50% of this population responded positively to questions 19, 27, and 29 (of which, 19 and 27 are part of the five diagnostic questions), which all represent dorsal stream function. Whilst each question will not be equally likely to elicit a positive response amongst a typical population, it is clear that the most common behavioural features amongst this cohort are related to dorsal stream function. It is more common to find dysfunction of the dorsal stream with an intact ventral stream (Dutton, 2010) but the pattern of responses shown in this study may expose specific impairments related to children with DS.

The use of the diagnostic 5 questions divides the data into two normal distributions (see Figure 1), with considerable overlap but different means. This raises the question as to whether the five-question screening tool is appropriate for children with DS or whether alternative questions may result in a more precise distinctions. For example, there are grounds to consider excluding question 27, which elicited a positive response from most participants, and which may represent a characteristic of learning disability in this population. On the other hand, five questions were scored positively only for children with suspected CVI, including ones relating to face recognition. It has already been observed that isolated ventral stream dysfunction is rare and is often combined with difficulties relating to dorsal stream dysfunction (Dutton, 2010). A particular deficit in processing faces in children with DS has been previously described (Wishart and Pitcairn, 2000). The findings in this study suggest that this difficulty with facial recognition may be part of a wider range of impairments relating to CVI. However, the numbers identifying poor face recognition were very low, so these and the other exclusive questions may not be suitable for a screening tool specific to children with DS. Further research is clearly needed to explore identification of a deficit of CVI origin and not related to other impairments, such as learning disability or mobility.

### Gender

Male predominance has previously been recognised amongst the DS population and the male to female sex ratio in children with DS has been reported as 1.23 (Kovaleva et al., 2001). Although the current analysis sample has an uncharacteristically high proportion of females, there does not appear to be any gender bias in responders and gender does not appear to be influential on suspected CVI outcome.

### Optometric Measurements

The relatively high prevalence of nystagmus, reduced acuity, strabismus and accommodative deficits reported here are consistent with other studies. Analysis showed that none of these functions was predictive of suspected CVI and they are therefore unlikely to be causal factors of the behavioural features. A recent study of children with a diagnosis of CVI and with a variety of risk factors (not including DS) reported that almost half had normal visual acuity (Sakki et al., 2021), confirming that CVI should never be assumed to be limited to children with poor acuity (Dutton, 2021).

Longitudinal studies have demonstrated a failure of the emmetropisation process in children with DS and that large refractive errors tend to either remain stable or increase with age (Haugen et al., 2001; Cregg et al., 2003) in young children, although no large changes to the spherical component of refractive error occur over the age of 4 years (Al-Bagdady et al., 2011). Thus, refractive errors in this study's population of 4–17-year-olds can be considered stable and this was confirmed by Pearson correlation.

Absolute refractive error was the only optometric factor to be significantly different between those with and without suspected CVI. The odds of screening positive for CVI increase by approximately 40% per dioptre of absolute spherical equivalent and children with over 5D of refractive error are more likely to screen positive for CVI than not. However, a model based on refractive error increases the likelihood of correctly predicting suspected CVI by only a small amount. The association between CVI and refractive error among other groups of children does not appear to have been investigated.

### Limitations

Although effort was made to select a random sample, some level of bias is likely present since all invited children were existing patients of Cardiff University's Special Assessment Clinic and may have already been experiencing some sort of visual difficulty. Information such as ethnicity, level of education and income were not collected. No participant had a diagnosis of brain injury, but childhood medical history was not obtained; this could be informative in terms of exposing the underlying cause of behavioural features, such as subtle oxygen deprivation.

### Implications

It is clear that visual perceptual difficulties are common amongst children with Down syndrome and that further work needs to be done to understand the origin of the difficulties. Visual perception difficulties have been identified as a potential cause for academic underachievement (Williams et al., 2011), and as children with DS are considered visual learners (Yang et al., 2014) recognition of possible CVI is important in ensuring that these children can access education tailored to their requirements (Dutton, 2021). The findings in the current study would suggest that generally, children with DS tend to exhibit more problems with visual perception than might be expected.

Cognitive impairment is a characteristic of Down syndrome and many of the problems occurring in children both with and without suspected CVI may be attributable to the cognitive impairment. However, the impact of CVI on performance on tests of cognitive ability, which often involve the use of visual attention, spatial orienting, visual perception and visual motor skills (Moore et al., 2002) has not, to our knowledge, been explored. If CVI is present and unrecognised, it may be that a child's cognitive function is at risk of being underestimated.

### General Conclusions and Summary

The majority of children with DS aged 4–17 years in this study experienced some level of visual perceptual difficulty and 38.3% met the screening criteria for suspected CVI.

Whilst children with DS are known to have a high prevalence of visual deficits, this study has shown that only refractive error is an indicator of the likelihood of CVI-related behavioural features. It appears likely that CVI is the explanation for the frequent visual perception impairments in children with DS and that further investigations are warranted. Optometric deficits are unrelated conditions that often coincide within this group.

Further research is clearly warranted into the aetiology of the visual perceptual problems that appear so prevalent in children with DS, and the likelihood of a diagnosis of CVI. According to Sakki et al. (2021) “the economic, social, and personal burden of CVI is high, with adverse effects of coexisting disorders increasing the burden further.” _It is essential that visual problems associated with CVI are explored in the assessment of children with DS, and that difficulties are not simply considered to be due to the learning disability or to inappropriate behaviour. Targeted strategies can be helpful in ameliorating the effects of CVI (McKillop and Dutton, 2008; Tsirka et al., 2020), and these should be considered for any child with DS who exhibits difficulties.

## Data Availability Statement

The raw data supporting the conclusions of this article will be made available by the authors, without undue reservation.

## Ethics Statement

The studies involving human participants were reviewed and approved by the National Institute for Social Care and Health Research Ethics Service (08/MRE09/46, amendment 5, 7th July 2016). Written informed consent to participate in this study was provided by the participants' legal guardian/next of kin.

## Author Contributions

JMW supervised the study and contributed to the write-up. GW carried out substantial data collection and did the bulk of the analysis and write-up. RW acted as statistical advisor for the study, did some of the statistical analysis, and contributed to the write-up. VV-N devised the methodology, carried out a proportion of the data collection, and contributed to the write-up. RE carried out a proportion of the data collection and contributed to the write-up. All authors contributed to the article and approved the submitted version.

## Conflict of Interest

The authors declare that the research was conducted in the absence of any commercial or financial relationships that could be construed as a potential conflict of interest.
